# Frontal Lobe Neurocysticercosis Presenting With Schizoaffective Symptoms in an Adolescent Immigrant

**DOI:** 10.7759/cureus.92071

**Published:** 2025-09-11

**Authors:** Emmanuel Annor, Grace Lee, Sam Kelly, Joseph Venditto, Elizabeth Mutter

**Affiliations:** 1 Child and Adolescent Psychiatry, Lehigh Valley Health Network, Allentown, USA; 2 Psychiatry, University of South Florida Morsani College of Medicine, Tampa, USA; 3 Psychiatry, Lehigh Valley Health Network, Allentown, USA

**Keywords:** antipsychotic and antiparasitic treatment, immigrant health, neurocysticercosis (ncc), psychiatric manifestations, seizures and neurological complications

## Abstract

Neurocysticercosis (NCC), caused by the larval stage of *Taenia solium*, is a parasitic infection of the central nervous system (CNS) that is increasingly identified in non-endemic regions due to global migration, while intraventricular and subarachnoid manifestations, especially those associated with frontal lobe involvement, remain underrecognized. This case report describes a 16-year-old immigrant who presented with psychotic and mood symptoms consistent with schizoaffective disorder, including hallucinations, suicidal ideation, and behavioral dysregulation managed by agitation. Neuroimaging revealed a right frontal lobe cyst with surrounding edema. The lesion with location and vesicular phase informed a treatment plan involving albendazole, corticosteroids, and long-acting antipsychotics. The patient's psychotic symptoms improved significantly following antiparasitic therapy and initiation of long-acting antipsychotic treatment. This case highlights the neuropsychiatric impact of frontal lobe NCC and underscores the importance of integrating neuroimaging, psychiatric evaluation, and targeted treatment in immigrant populations presenting with atypical psychiatric syndromes.

## Introduction

Neurocysticercosis (NCC), caused by the larval stage of *Taenia solium*, remains a leading parasitic infection of the central nervous system (CNS) in endemic regions. Transmission occurs through the ingestion of eggs from contaminated feces or the consumption of undercooked pork containing cysticerci. While pigs serve as intermediate hosts, humans may develop intestinal taeniasis or cysticercosis depending on the route of exposure. Once in the CNS, cysticerci can persist in a vesicular form for years, particularly when host immunity is permissive [1].

Clinical manifestations vary by lesion location. Parenchymal NCC commonly presents with seizures, while extraparenchymal involvement may lead to intracranial hypertension or obstructive hydrocephalus. Neuroimaging remains the diagnostic gold standard. Magnetic resonance imaging (MRI) excels in identifying viable cysts and extraparenchymal disease, whereas computed tomography (CT) is more sensitive for detecting calcified lesions. Serological assays, such as enzyme-linked immunoelectrotransfer blot (EITB), offer high specificity in cases with multiple viable cysts; however, enzyme-linked immunosorbent assay (ELISA) tests are less reliable for single or calcified lesions [1,2].

Treatment decisions depend on the type, number, and location of the lesions, guided by recommendations from the Infectious Diseases Society of America (IDSA) and the American Society of Tropical Medicine and Hygiene (ASTMH). Albendazole (15 mg/kg/day for 10-14 days) is standard for viable parenchymal lesions, with praziquantel (50 mg/kg/day) reserved for extensive or extraparenchymal disease. Corticosteroids are co-administered to mitigate inflammatory responses during antiparasitic therapy. Surgical intervention is indicated for intraventricular cysts or obstructive hydrocephalus; however, the evidence supporting these approaches varies in strength [3].

NCC is endemic in Asia, sub-Saharan Africa, and Latin America, where poverty and poor sanitation facilitate transmission. Diagnostic limitations, particularly restricted access to neuroimaging and reduced serological sensitivity in calcified lesions, contribute to underreporting. Asymptomatic cases often go undetected, and NCC accounts for up to 30-70% of epilepsy in endemic populations [4]. In the United States, NCC is rare but increasingly recognized due to global migration, with over 2,300 hospitalizations annually [5].

Neuropsychiatric symptoms in NCC, particularly those arising from frontal lobe involvement, are underexplored and often based on observational data or expert consensus. Frontal lobe cysts may disrupt executive function, mood regulation, and affective stability, contributing to schizoaffective presentations marked by mood-congruent psychosis, agitation, and cognitive disorganization. Approximately 15% of patients in endemic areas exhibit cognitive dysfunction, depression, anxiety, or personality changes. Psychotic features may include confusion, delusions, aggression, and visual hallucinations, often linked to inflammatory disruption of neural circuits [6].

This case presentation examines frontal lobe NCC manifesting with schizoaffective symptoms in an adolescent immigrant, highlighting diagnostic complexities, neuropsychiatric management, and implications for long-term functional recovery.

## Case presentation

A 16-year-old refugee from sub-Saharan Africa was referred to the emergency department (ED) following an episode of brief unresponsiveness and staring. His mother reported severe headaches the day prior. The patient had no prior history of seizures, head trauma, or CNS infections and was up to date on vaccinations. The first seizure reportedly occurred approximately a few months after arrival in the United States. There was no family history of epilepsy.

The patient displayed alertness and activity during the ED examination, with physical findings appearing unremarkable. A focused mental status examination indicated no abnormalities; the patient was oriented to person, place, and time, exhibited an appropriate mood and affect, demonstrated a coherent thought process, and showed no perceptual disturbances.

**Figure 1:**
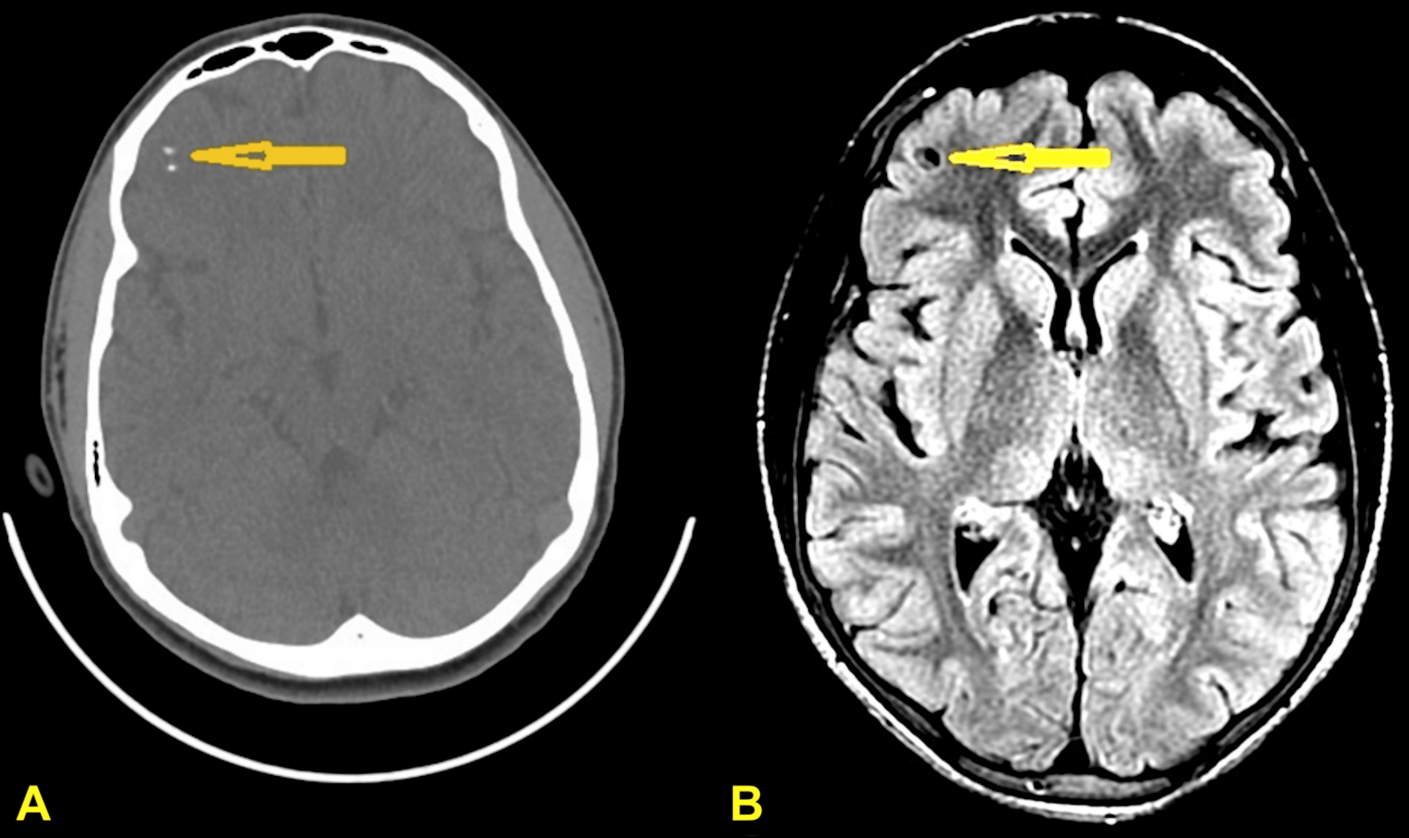
CT and MRI imaging of neurocysticercosis (A) Initial CT scan reveals calcifications within the right frontal lobe, consistent with chronic or inactive neurocysticercosis. (B) Initial MRI (T2-weighted and FLAIR sequences) identifies a 6.1 mm cystic lesion in the right frontal subcortical region, with a visible scolex along the wall—features consistent with a vesicular-stage lesion. These imaging findings fulfill the major neuroimaging criteria for neurocysticercosis, as defined in established diagnostic frameworks. FLAIR: fluid-attenuated inversion recovery; MRI: magnetic resonance imaging; CT: computed tomography

MRI of the brain, including T1-weighted, T2-weighted, FLAIR (fluid-attenuated inversion recovery), and contrast-enhanced sequences, revealed a 6.1 mm cystic lesion in the right inferior lateral frontal lobe with a visible scolex. The lesion was hyperintense on T2-weighted images and hypointense on FLAIR images. CT imaging showed calcifications in the same region (Figure 1). These findings were reviewed and confirmed by neurology and neuroradiology, who staged the lesion as viable and vesicular.

Laboratory studies indicated that the patient had microcytic anemia, thrombocytopenia, low ferritin levels, and low levetiracetam levels. The infectious workup included tuberculosis QTF, strongyloides antibody, stool ova and parasite testing, and cysticercosis IgG serology. The cysticercosis test was performed using an ELISA method, which returned a negative result (index value of 3, below the diagnostic threshold of 8) (Table 1).

**Table 1:** Initial laboratory workup Blood tests revealed microcytic anemia, thrombocytopenia, and low ferritin levels. The results for cysticerci IgG, *Strongyloides* antibody, tuberculosis, and stool test were all within normal limits.

Parameters	Patient Values	Reference Value
Complete Blood Count		
Hemoglobin	11.4	11.9–14.8 g/dL
White Blood Count	4.3	3.8–10.4 thou/cmm
Platelets	127	158–362 thou/cmm
Mean Corpuscular Volume	60	83–98 fl
Mean Corpuscular Hemoglobin	19.2	27.6–33.3 pg
Mean Corpuscular Hemoglobin Concentration	31.8	32.5–35.2 g/dL
Red Cell Distribution Width	19.4	11.4–13.5%
Complete Metabolic Panel		
Sodium	136	135–145 mmol/L
Potassium	4.2	3.5–5.2 mmol/L
Calcium	9.6	8.5–10.1 mg/dL
Chloride	105	100–109 mmol/L
Creatinine	0.56	0.50–0.80 mg/dL
Blood Urea Nitrogen	11	7.0–20 mg/dL
Carbon Dioxide	25	21–31 mmol/L
Glucose	92	65–99 mg/dL
Liver Function Test		
Albumin	4	3.8–5.2 g/dL
Protein, Total	6.9	6.2–7.7 g/dL
Total Bilirubin	0.3	0.2–0.8 mg/dL
Aspartate Aminotransferase	36	12.0–24.0 U/L
Alanine Aminotransferase	16	< 56 U/L
Alkaline Phosphatase	269	52.0–239 U/L
Differentials		
Tuberculosis QTF	Negative	Negative
*Strongyloides* Antibody	0.5	< 0.9 IV
Ova/Parasites, Stool	No organisms observed	Negative
Cysticercosis IgG	3	< 8 IV
Ferritin	6	11–172 ng/mL
Levetiracetam	< 1.0	3.1–10.0 mcg/mL
Drug Screen, Urine	Negative	Negative

A sleep-deprived EEG was performed to evaluate for subclinical seizure activity and further characterize the seizure disorder. The EEG showed normal awake, stage I, and stage II sleep patterns, with no epileptiform discharges.

Despite the negative serology, imaging findings, and epidemiological context, the diagnosis of NCC was supported. Differential diagnoses included tuberculoma, brain abscess, mycotic granuloma, cystic echinococcosis, coenurosis, and brain tumor.

Initial seizure management included a loading dose of levetiracetam (20 mg/kg), followed by a maintenance dose of 1000 mg twice daily according to pediatric management protocols. Ibuprofen and acetaminophen were prescribed to alleviate headaches. The next day, he developed acute agitation and aggression. Delirium was considered but less likely. Levetiracetam was discontinued due to behavioral side effects, and haloperidol was initiated. Lorazepam was administered intramuscularly and intravenously for sedation. A 2:1 observation ratio was implemented. Fosphenytoin was given as a loading dose, followed by phenytoin 100 mg twice daily. A breakthrough seizure on day four prompted a second loading dose and an increase to 130 mg twice daily.

Treatment for NCC was initiated after obtaining ophthalmologic clearance and confirming normal intracranial pressure. Fundus examination showed no papilledema or intraocular cysts. Dexamethasone (0.1 mg/kg BID) was started prior to albendazole (400 mg BID for 10-14 days).

During hospitalization, the patient expressed suicidal thoughts. A structured psychiatric assessment using the Columbia-Suicide Severity Rating Scale (C-SSRS) led to suicide precautions, which were lifted following clinical improvement. At discharge, the patient was prescribed phenytoin 130 mg daily and haloperidol 5 mg daily, with 5 mg every eight hours as needed for agitation. Haloperidol was continued due to its effectiveness.

Over the following months, the patient had six ED visits for seizures, altered mental status, aggression, and hallucinations (Table 2). His regimen was adjusted to risperidone (1 mg BID), and phenytoin was increased to 200 mg BID.

**Table 2:** Summary of patient ED presentations after discharge ED: emergency department; PICU: pediatric intensive care unit; IM: intramuscular; EMS: emergency medical services

ED Visit	Reason Presentation
Visit 1	Aggression - Unprovoked behavioral escalation.
Visit 2	Agitation - Self-presented to ED without identifying information, citing external distress.
Visit 3	The patient exhibited agitation following a sibling's PICU hospitalization. Security intervention and pharmacologic management with IM haloperidol and midazolam were required, followed by four-point restraints.
Visit 4	The patient attempted to leave home; the father intervened, leading to escalated behavior. Law enforcement was contacted for assistance.
Visit 5	The patient was transported via EMS following an incident involving the possession of a knife near a family member. No injuries reported.
Visit 5	The patient initially presented with altered mental status, later exhibiting recurrent episodes of agitation in the context of inconsistent medication adherence.

Five months after discharge, the patient was hospitalized due to escalating anger and aggression. During the admission, he endorsed persistent hopelessness, auditory hallucinations, and suicidal ideation. The mental status examination revealed diminished alertness and a marked preoccupation with internal stimuli. His mood was dysphoric, and his affect was labile and incongruent, with inappropriate laughter and smirking while discussing suicidal thoughts and paranoid beliefs. His speech was spontaneous and coherent, although his thought process was disorganized and paranoid in nature. He was actively responding to auditory hallucinations. Orientation was intact, but attention and concentration were poor, and his fund of knowledge appeared limited. Judgment and insight were significantly impaired.

In addition to the mental status examination, standardized assessments were used to support diagnostic clarity. During the acute phase, the Brief Psychiatric Rating Scale (BPRS) indicated moderate to severe psychotic symptoms, and the C-SSRS confirmed active suicidal ideation.

Given the co-occurrence of mood symptoms, psychotic features, and cognitive deficits, alongside a known history of frontal lobe NCC, the clinical picture was consistent with schizoaffective disorder, depressed type. The diagnosis was made in light of the patient's affective instability, persistent depressive symptoms, and psychosis occurring outside of mood episodes. Chronic brain changes from NCC were considered a likely contributing factor to the emergence and persistence of these symptoms, particularly given the lesion's location and prior neuropsychiatric sequelae.

Phenytoin was continued at 200 mg BID. Due to ongoing nonadherence, risperidone was replaced with long-acting injectable paliperidone palmitate. A loading dose of 234 mg was administered, followed by a dose of 156 mg and monthly maintenance doses of 156 mg. No significant side effects were reported.

**Figure 2:**
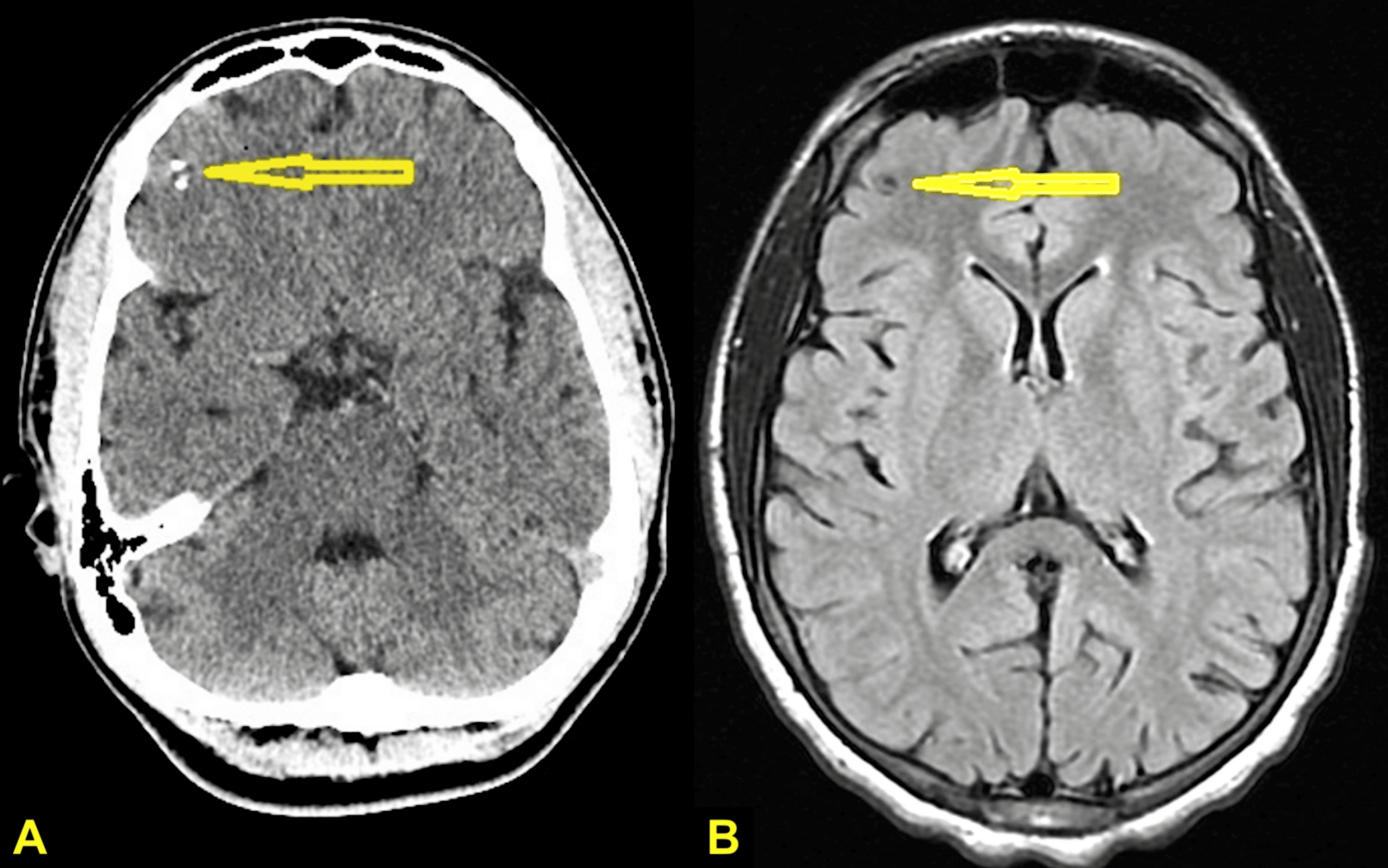
(A) A follow-up CT scan reveals a stable cystic lesion with partial calcification in the right frontal lobe. (B) An MRI from the same period confirms no significant changes, with a persistent stable cystic lesion in the right lateral frontal lobe

The patient remained stable on paliperidone palmitate, with a marked reduction in ED visits from six in five months to one in the two months following stabilization. He continues phenytoin and has intranasal diazepam for prolonged seizures. Follow-up MRI and CT scans showed no change in lesion size or surrounding edema (Figure 2). Imaging was reviewed by the same neuroradiologist for consistency. Given the stability of the right frontal lesion, ongoing neuroinflammatory activity was considered less likely. The patient's psychiatric symptoms were therefore interpreted as more consistent with sequelae of chronic brain injury.

## Discussion

Our case demonstrates a compelling association between frontal lobe NCC and the emergence of severe schizoaffective symptoms in a previously psychiatrically healthy adolescent refugee from sub-Saharan Africa. The patient initially presented with brief unresponsiveness and staring, progressing rapidly to complex neuropsychiatric manifestations including auditory hallucinations, suicidal ideation, aggressive behavior, and mood dysregulation. Neuroimaging revealed a 6.1 mm cystic lesion in the right inferior lateral frontal lobe with a visible scolex, staged as viable and vesicular on MRI sequences (Figure 1). Despite negative serological testing for cysticercosis IgG (index value of 3, below the diagnostic threshold of 6) as shown in Table 1, the diagnosis was supported by characteristic imaging findings and epidemiological context. This presentation aligns with established literature demonstrating that frontal lobe involvement in NCC can disrupt executive function, mood regulation, and affective stability, contributing to complex psychiatric presentations [6,7]. The temporal relationship between lesion identification and psychiatric decompensation, combined with the patient's demographic profile as an immigrant from an endemic region, strongly supports the contributory role of NCC in his neuropsychiatric syndrome.

The clinical course was marked by significant behavioral volatility and multiple emergency department presentations, as detailed in Table 2. Following initial seizure management with levetiracetam, the patient developed acute agitation and aggression, requiring discontinuation of the antiepileptic due to behavioral side effects. The progression from isolated seizure activity to complex psychiatric symptoms mirrors patterns described in previous case reports of NCC-associated psychosis [8,9]. Forlenza et al. documented similar psychiatric manifestations in their study of 38 patients with NCC, noting that frontal lobe lesions were particularly associated with behavioral disturbances and mood instability [7]. Our patient's presentation of pressured speech, disorganized thought processes, active auditory hallucinations, and paranoid ideation during acute phases closely resembles the psychotic profiles reported in comparable cases. The BPRS indicated moderate to severe psychotic symptoms during the acute phase, while the C-SSRS confirmed active suicidal ideation, necessitating intensive psychiatric management alongside antiparasitic therapy.

Treatment challenges were multifaceted, encompassing both the underlying parasitic infection and the emergent psychiatric complications. Following ophthalmologic clearance confirming normal intracranial pressure, standard antiparasitic therapy was initiated with albendazole 400 mg twice daily for 14 days, preceded by dexamethasone 0.1 mg/kg twice daily to mitigate inflammatory responses [3]. However, medication adherence proved problematic, contributing to repeated emergency presentations for agitation, altered mental status, and aggressive behavior. Similar adherence challenges have been documented in other NCC cases with psychiatric comorbidity, particularly in immigrant populations facing language barriers and cultural adaptation stressors [10,11]. Mishra and Swain reported comparable difficulties in their case series, emphasizing that psychiatric manifestations following NCC often require prolonged, multidisciplinary management approaches [10]. The patient's recurrent episodes of aggression and behavioral escalation, including incidents requiring law enforcement intervention and emergency medical transport, underscore the complexity of managing NCC-related neuropsychiatric symptoms in vulnerable populations.

The broader implications of this case extend beyond individual patient management to highlight systemic challenges in diagnosing and treating NCC-related psychiatric presentations in immigrant populations. The patient's demographic profile as a refugee from sub-Saharan Africa, combined with the characteristic imaging findings and clinical presentation, exemplifies the increasing recognition of NCC in non-endemic regions due to global migration patterns [4,5]. The diagnostic challenges are further compounded by the diverse psychiatric presentations studied for the case, ranging from bipolar disorders with cyclical manic and depressive episodes [8-10] to psychosis with various manifestations [11-14]. The limitations of serological testing in single-lesion NCC, as demonstrated by our patient's negative cysticercosis IgG despite a viable lesion on imaging, reinforce the critical importance of neuroimaging and clinical context in diagnosis [1,2]. Laboratory abnormalities, including microcytic anemia, thrombocytopenia, and low ferritin levels (Table 1), likely reflected nutritional deficiencies common in refugee populations rather than NCC-specific findings. The successful long-term stabilization achieved with paliperidone palmitate, combined with continued anticonvulsant therapy, demonstrates the potential for favorable outcomes when comprehensive, culturally sensitive care models are implemented.

This case underscores the need for heightened clinical suspicion of NCC in immigrants presenting with new-onset psychiatric symptoms, particularly when accompanied by seizures or neurological findings, and supports the growing literature advocating for interdisciplinary approaches to managing the complex neuropsychiatric sequelae of this preventable but persistent global health challenge.

## Conclusions

This case highlights a plausible association between frontal lobe NCC and the emergence of schizoaffective symptoms in an adolescent immigrant with no prior psychiatric history. While causality cannot be definitively established from a single case, the temporal proximity of lesion identification, seizure onset, and psychiatric decompensation, combined with partial symptom resolution following antiparasitic and psychiatric treatment, suggests a contributory role of NCC in the patient's neuropsychiatric presentation. The use of standardized psychiatric assessments and comparative case data strengthens the clinical narrative and supports further exploration of NCC's psychiatric impact. Future studies should investigate this relationship in larger cohorts to clarify mechanisms and guide interdisciplinary management.
